# Behavior problems and personality in Korean high school students

**DOI:** 10.7717/peerj.6106

**Published:** 2018-12-10

**Authors:** Soo Jin Lee, Soo Hyun Park, C Robert Cloninger, Han Chae

**Affiliations:** 1 Department of Psychology, Kyungsung University, Busan, South Korea; 2 Department of Psychology, Yonsei University, Seoul, South Korea; 3 Department of Psychiatry, School of Medicine, Washington University in St. Louis, St. Louis, MO, United States of America; 4 School of Korean Medicine, Pusan National University, Busan, South Korea

**Keywords:** Temperament and character inventory, Behavior problem, Youth self-report, Latent profile analysis, Adolescents, Matured personality

## Abstract

**Introduction:**

Extant studies have examined the effect of psychological characteristics on clinical features that define behavior problems. The purpose of this study was to analyze the effects of temperament and character as both individual factors and complex profiles on behavior problems in a community sample of adolescents.

**Methods:**

Behavior problems and personality of 670 Korean high school students were measured with the Youth Self-Report (YSR) and Junior version of the Temperament and Character Inventory (JTCI). Stepwise regression analysis analyzed the effects of JTCI character and temperament traits on YSR Total, Internalizing and Externalizing subscale scores, and Profile Analysis examined differences of JTCI personality profiles among three latent YSR subscale profiles acquired from Latent Profile Analysis.

**Results:**

Seven subscales of the JTCI explained 38% of the YSR total degree of behavior problems, and JTCI Novelty-Seeking and Harm-Avoidance were found to account for vulnerability while JTCI Reward-Dependence and Self-Directedness explained resilience to behavior problems. There were three distinct latent YSR profile groups based on nine YSR subscales, and low behavior problem group showed a resilient personality profile characterized by low Novelty-Seeking and Harm-Avoidance and high Reward-Dependence, Persistence, Self-Directedness and Cooperativeness while high behavior problem group exhibited a vulnerable personality profile of the opposite tendency.

**Discussion:**

Temperament and character explained behavior problems of Korean high school students as both individual personality traits and a complex personality profile. The results and implications of this study were examined in regard to mental health of adolescents, and the importance of education in the development of mature personality are discussed.

## Introduction

Adolescence is a developmental period characterized by rapid growth of both body and mind, and establishment of psychological identity which may determine lifespan mental health through the process of interaction with society (Klimstra et al., 2010). And adolescents are at particular risk of developing behavior problems resulting from daily life stress as they adjust themselves as members of society. The behavior problems of Korean adolescents experiencing academic stress and rapid sociocultural changes have been a major issue (Oh & Lee, 1990), and various studies attempting to delineate this phenomena were conducted (Kim, Lee & Chae, 2017; Lee et al., 2017b; Lee et al., 2013).

Behavior problems demonstrate typical clinical characteristics depending on gender, age and developmental stage, and such complex clinical spectrums need to be measured from a wide-band perspective rather than focusing on specific problems (Oh & Lee, 1990). The Achenbach System of Empirically Based Assessment (ASEBA) (Achenbach, 2009; Achenbach & Rescorla, 2001), which was developed by Achenbach and Edelbrock (Achenbach & Edelbrock, 1978), provides clinical assessment of behavior problems in diverse age groups using a dimensional approach to understand the cause and maintenance of behavior problems (Achenbach, 2009).

The ASEBA categorizes behavior problems into three groups, namely Internalizing Problems, Externalizing Problems and Total Problems (Achenbach, 2009). The Internalizing Problems of Anxious/Depressed, Withdrawn/Depressed and Somatic Complaints are deemed to result from excessive internal control of emotional problems, and are reported to occur more frequently in females (Nolen-Hoeksema & Girgus, 1994; Rutter, Caspi & Moffitt, 2003). The Externalizing Problems refer to Rule-Breaking and Aggressive Behavior sought to represent excessive expression of psychological difficulties, and has been shown to be more prevalent in males (Archer, 2004; Else-Quest et al., 2006). The Total Problems is the sum of Internalizing and Externalizing Problems along with Social Problems, Thought Problems, Attention Problems and Other Problems.

Several previous studies showed that specific psychological traits may be useful in effective screening and preventive intervention of particular behavior problems (Caprara et al., 2017; Cooper et al., 2003; De Pauw, Mervielde & Van Leeuwen, 2009; Hoyle, 2000). As for vulnerability and resilience, the Five Factor Model (Costa & MacCrae, 1992) suggested that Internalizing Problems are associated with low extraversion and high neuroticism, and that Externalizing Problems are related to high extraversion, low agreeableness, and low conscientiousness (Slobodskaya & Akhmetova, 2010).

However, since the Five Factor Model is based on factor analysis of psychological characteristics of an individual (Costa & MacCrae, 1992), it holds limitations in that only mixture of expressed traits are measured without distinguishing relatively hereditary and stable temperament traits from acquired and changeable character traits across the lifespan. We need a biopsychosocial measure of the temperament facets along with character aspects which may be influenced by the environment and learning (Lee et al., 2014) because it may provide the theoretical grounds for intervention aimed at prevention and treatment of behavior problems (Kim, Lee & Chae, 2017; Lee et al., 2017b; Lee et al., 2013).

The Temperament and Character Inventory (TCI), a personality assessment tool based on Cloninger’s biopsychosocial personality model (Cloninger, 1987; Cloninger, Svrakic & Przybeck, 1993), consists of two domains measuring temperament and character, respectively, and explains the foundation and development of personality through their interaction.

Temperament, encompassing Novelty Seeking (NS), Harm Avoidance (HA), Reward Dependence (RD), and Persistence (PS), is an innate and biological foundation for character development based on the behavioral activation and inhibition system proposed by Gray (Gray, 1982) and the behavior maintenance system suggested by Sjobring (Sjobring, 1973). Character, comprised of Self-Directedness (SD), Cooperativeness (CO), and Self-Transcendence (ST), represents the value of the person regarding him/herself, society and universe, and is developed through interactions between the temperament and the environment (Cloninger, 1987; Cloninger, Svrakic & Przybeck, 1993).

Previous studies have reported correlations between personality traits and a wide spectrum of behavior problems using the ASEBA and Junior version of the TCI (JTCI). The HA temperament was shown to have a strong positive correlation with Internalizing Problems such as depression and anxiety (Copeland et al., 2004; Hemphälä, Gustavsson & Tengström, 2013; Kim, 2013; Kim et al., 2006; Lim, Choi & Choi, 2013; Rettew et al., 2006), while NS and RD temperaments (Cloninger, Svrakic & Przybeck, 1993; Kim et al., 2006; Lim, Choi & Choi, 2013) and SD character (Copeland et al., 2004; Kim, 2013; Kim et al., 2006) showed negative correlations with depression and anxiety. The NS temperament alone (Copeland et al., 2004; Ha & Kwon, 2016; Hemphälä, Gustavsson & Tengström, 2013; Kim & Lee, 2016; Kim et al., 2006; Rettew et al., 2006) or in association with HA (Kim et al., 2006) demonstrated a positive correlation with Externalizing Problems such as violence and rule-breaking, and the SD and CO characters (Copeland et al., 2004; Kim et al., 2006) showed a negative correlation.

However, the human personality is a complex adaptive system comprised of multiple dimensions interacting non-linearly, rather than a collection of individual components (Cloninger, 2008). Clinicians and researchers need a profile analysis providing person-centered perspectives appropriate for detecting such non-linear and complex dynamics of personality (Cloninger & Cloninger, 2011; Cloninger & Zohar, 2011), not only focusing on the average effects of separate traits in regard to vulnerability and resilience across groups of individuals (Cloninger et al., 2012b). Since healthy, resilient, and matured personality profile of adults was characterized by low HA and high SD and CO in previous studies (Cloninger & Cloninger, 2011; Cloninger & Zohar, 2011; Eley et al., 2013; Eley et al., 2016), this possibility must also be examined and confirmed in adolescents (Moreira et al., 2014).

For such reasons, we recruited a community sample of Korean adolescents, and analyzed the effects of temperament and character as both individual factors and complex profiles on behavior problems using the JTCI and Youth Self Report (YSR) (Oh & Kim, 2011; Oh & Lee, 1990). We used stepwise multiple regression to examine whether temperament and character traits explain behavior problem patterns, and also performed latent profile analysis (Eley et al., 2016; Lee, Choi & Chae, 2017a) to uncover personality profiles related to specific severity of behavior problems with consideration of gender (Moreira et al., 2014; Oh & Lee, 1990; Pursell et al., 2008) which has not been reported previously.

Adolescents are able to report their behavior problems, especially internalizing problems more reliably than primary caregivers and the development of their personality expands in this developmental period to play a pivotal role as a protective factor of psychological health in adulthood. Korean high school students who have relatively developed personalities and who can complete validated self-report measures (Sourander, Helstelä & Helenius, 1999; Verhulst & Ende, 1992) would be fit for this study as such findings may be generalizable to preventive interventions in the future (Roberts, Caspi & Moffitt, 2001).

This study may contribute to person-centered research and theory related to the correlation between psychopathology and complex personality characteristics in line with previous studies of vulnerable and resilient personality factors (Eley et al., 2016; Lee, Choi & Chae, 2017a; Moreira et al., 2014) which showed the importance of nurturing matured personality for coping with daily life stress.

## Methods and Materials

### Subjects

Korean high school students between the ages of 16 to 18 from the Daegu metropolitan area voluntarily participated in the present study as part of their extracurricular activity. Behavior problems and personality traits of 686 students were measured and data of 16 subjects were excluded due to incompletion of questionnaires. Subsequently, a total of 670 participants were included in the study. This study was approved by the Internal Review Board (2014/11/26-001), and all participants provided written informed consent for this study.

### Youth Self-Report (YSR)

The adolescents’ behavior problems were measured using the Korean version of the Youth Self Report (YSR) for ages 12–17 (Oh & Kim, 2011), a questionnaire including 118 items developed to measure the behavior problem in adolescents. YSR is originated from Achenbach System of Empirically Based Assessment (ASEBA)(Achenbach, 2009; Achenbach & Rescorla, 2001). Adolescents were recruited to rate the behavior problems for the past six months with a three-point Likert scale (0, not true at all; 1, somewhat or sometimes true; and 2, very true or often true). The Korean version of YSR was standardized and validated in 2011 and demonstrated good validity and reliability (Oh & Kim, 2011).

The YSR yields three subscales consisting of Total score, Internalizing Problems and Externalizing Problems, and eight clinically validated symptom clusters. Internalizing Problems has Anxious/Depressed, Withdrawn/Depressed, and Somatic Complaints and Externalizing Problems behavior has Rule-Breaking Behavior and Aggressive Behavior. The Total Problems is a sum of Internalizing Problems, Externalizing Problems, Social Problems, Thought Problems, Attention Problems and Other Problems. Total, Internalizing, Externalizing Problems and five empirically validated symptom clusters related to Internalizing and Externalizing Problems were analyzed in the present study. Cronbach’s alphas for the Total, Internalizing, and Externalizing Problems were 0.78, 0.75, and 0.90, respectively, in the current study.

### Junior version of the Temperament and Character Inventory (JTCI)

Cloninger’s Temperament and Character Inventory (TCI) consist of two-interrelated dimensions of temperament and character. Temperament traits depict biases in automatic responses to emotional stimuli including involuntary rational processes, while character traits reflect differences in higher cognitive functions related to values, goals, and relationships of a person (Cloninger, 2008; Cloninger, Svrakic & Przybeck, 1993).

The temperament dimension includes Novelty Seeking (NS, characterized by exploratory excitability, extravagance, disorderliness, and impulsiveness) associated with exploratory excitability in response to novel stimuli, Harm Avoidance (HA, anticipatory worry, shyness with strangers, fear of uncertainty, and fatigability) related with behavioral inhibition in response to dangerous or abhorrent stimuli, Reward Dependence (RD, attachment, sentimentality, openness, and dependence) referring to the tendency to respond particularly to signals of social reward, and Persistence (PS, eagerness, ambition, work-hardened, and perfectionism) representing a tendency to maintain behavior in spite of intermittent reinforcement (Chae et al., 2014).

The character dimension is comprised of Self-Directedness (SD, characterized by purposeful, responsible, resourceful, and self-accepting) indicating the tendency to identify the self as an autonomous individual, Cooperativeness (CO, empathic, helpful, forgiving, and tolerant) measuring the degree to which one considers the self as integral to humanity, and Self-Transcendence (ST, contemplating, idealistic, spiritual, and transpersonal) denoting the degree to which one considers the self as an integral part of the universe as a whole (Cloninger, 2008; Cloninger, Svrakic & Przybeck, 1993).

The Korean version of the Junior Temperament and Character Inventory (JTCI) is an 82-item self-report questionnaire for middle and high school students (ages 13 to 18), and asks individuals to score each item on a 4-point scale (0, not at all to 3, very true). It was standardized with acceptable validity and reliability in 2007. The internal consistencies of NS, HA, RD,PS, SD, CO, and ST were reported as 0.76, 0.81, 0.67, 0.67, 0.74, 0.71, and 0.66, respectively (Min, Oh & Lee, 2007).

### Statistical analysis

The significance of gender difference was examined using chi-square analysis for grade and *t*-tests for subscales of the JTCI and YSR. Pearson correlation analysis was used to examine the relationship between subscales of JTCI and YSR in male and female students.

Multiple stepwise regression analyses were conducted to examine the degree to which personality traits explained subscales of the YSR. First, age and gender were introduced (model 1), and then the seven JTCI subscale measure scores were added as the final model (model 2). The inclusion criterion for entering factors in the model was *p* value less than 0.05 associated with the *F*-statistic.

The Latent Profile Analysis (LPA) was employed to explore the latent groups of behavior problems and peronality in this study (Eley et al., 2016; Muthén & Muthén, 2005). The LPA is an empirically driven model extracting hidden groups of individuals based on similar chracteristics from observed continuous variables (e.g., nine subscales of YSR), and it estimates the probabilty of a particular observation falling into a specific class (Lanza, Flaherty & Collins, 2003). The number of classes is determined through comparison of posterior fit statistics: models are estimated with classes added repeatedly to determine which model is the best fit to the data until the addition no longer produces a significant improvement in model fit statistics. Gender was introduced as a covariate in LPA due to the gender difference in the current study.

The criteria of model fit in LPA were as follows (Nylund, Asparouhov & Muthen, 2007): (1) the smaller Bayesian Information Criterion (BIC) and adjusted BIC, the better the model is; (2) the smaller *p* value of Vuong-Lo-Mendell-Rubin Likelihood Difference Test (VLMR) or Lo-Mendell-Rubin Likelihood Difference Test (LMR), the better the model is; and (3) Entropy index was used to examine the distinctiveness of latent classes identified, assuming higher than 0.8 as good (Muthén, 2004).

After determining the number of latent classes in behavior problems, Analysis of Covariance (ANCOVA) and Profile Analysis were conducted with the nine YSR subscales and seven JTCI factors to elucidate significant differences among extracted latent groups from the LPA. We applied Bonferroni or Dunnett’s T3 for the post-hoc analysis depending on the results of Levene’s Homogeneity test with gender as the covariate, and the estimated value of subscales of the YSR and JTCI were also calculated with gender as a covariate. As for the Profile Analysis, flatness and parallelism were calculated, and Greenhouse-Geiser correction was incorporated when Mauchly’s sphericity test was significant.

The data are presented as means with standard deviations or frequencies with percentages. All analysis were performed using IBM SPSS Statistics 20.0 (IBM, Armonk, NY) and MPlus 5.21 (Muthen & Muthen, Los Angeles, CA) (Muthén & Muthén, 2005) and *p* values of 0.05, 0.01, and 0.001 were imployed for significance.

## Results

### Demographic features of the participants

The demographic characteristics of participants in this study are presented in Table 1. There were significant differences between male and female adolescents in regard to grade in high school (*χ*^2^ = 11.127, *p* < 0.004). As for the subscales of the JTCI, female students showed significantly higher RD, CO and ST scores than male students. As for the subscales of the YSR, female students demonstrated higher Internalizing Problems, Anxious/Depressed, and Somatic Complaints scores than male students, while male students reported higher Externalizing Problems and Rule-Breaking Behavior scores than female students.

**Table 1:** Demographic characteristics of participants.

		Male	Female	Total	
*N*		365	305		
Grade	Sophomore	128	115	243	*χ*^2^ = 11.127, *p* = 0.004
	Junior	116	123	239	
	Senior	121	67	188	
JTCI	NS	20.08 ± 5.77	20.54 ± 5.66	20.29 ± 5.72	*t* = − 1.043, *p* = 0.297
	HA	21.94 ± 6.77	22.61 ± 6.78	22.25 ± 6.78	*t* = − 1.287, *p* = 0.198
	RD^***^	15.31 ± 4.02	17.85 ± 4.06	16.46 ± 4.23	*t* = − 8.108, *p* < 0.001
	PS	13.48 ± 3.43	13.69 ± 3.29	13.57 ± 3.37	*t* = − 0.829, *p* = 0.408
	SD	21.99 ± 5.8	21.32 ± 5.99	21.69 ± 5.89	*t* = 1.468, *p* = 0.142
	CO^**^	25.95 ± 5.3	27.12 ± 4.98	26.48 ± 5.19	*t* = − 2.929, *p* = 0.004
	ST^***^	16.17 ± 4.81	17.81 ± 4.63	17.81 ± 4.63	*t* = − 4.457, *p* < 0.001
YSR	Total Problems	40.73 ± 24.45	40.09 ± 21.44	40.44 ± 23.11	*t* = 0.363, *p* = 0.717
	Internalizing Problems^*^	11.22 ± 8.78	12.91 ± 8.68	11.99 ± 8.77	*t* = − 2.497, *p* = 0.013
	Externalizing Problems^***^	10.56 ± 7.63	8.67 ± 5.76	9.7 ± 6.9	*t* = 3.666, *p* < 0.001
	Anxious/Depressed^**^	5.34 ± 4.36	6.38 ± 4.19	5.82 ± 4.31	*t* = − 3.134, *p* = 0.002
	Withdrawn/Depressed^*^	3.68 ± 3.29	3.22 ± 2.78	3.47 ± 3.08	*t* = 1.961, *p* = 0.05
	Somatic Complaints^***^	2.2 ± 2.6	3.31 ± 3.3	2.71 ± 2.99	*t* = − 4.776, *p* < 0.001
	Rule-Breaking Behavior^***^	3.71 ± 3.37	2.09 ± 2.21	2.97 ± 3.01	*t* = 7.469, *p* < 0.001
	Aggressive Behaviors	6.85 ± 4.88	6.58 ± 4.19	6.73 ± 4.57	*t* = 0.793, *p* = 0.428
	Social Problems	3.97 ± 3.49	3.82 ± 2.89	3.9 ± 3.23	*t* = 0.612, *p* = 0.541
	Thought Problems	4.22 ± 3.3	3.83 ± 2.77	4.04 ± 3.07	*t* = 1.674, *p* = 0.095
	Attention Problems	6.44 ± 3.61	6.56 ± 3.3	6.5 ± 3.47	*t* = − 0.444, *p* = 0.657
	Other Problems	4.32 ± 2.63	4.3 ± 2.55	4.31 ± 2.59	*t* = 0.067, *p* = 0.947

**Notes.**

* *p* < 0.05.

** *p* < 0.01.

*** *p* < 0.001.

JTCI Junior Temperament and Character Inventory YSR Youth Self-Report NS Novelty-Seeking HA Harm-Avoidance RD Reward-Dependence PS Persistence SD Self-Directedness CO Cooperativeness ST Self-Transcendence

### Correlation between subscales of the YSR and JTCI, and stepwise regression analysis for Total, Internalizing and Externalizing Problems with personality traits

The correlation coefficients between subscales of the YSR and JTCI were presented in Tables 2 through 4. NS and HA were positively correlated with Total Problems, Internalizing Problems and Externalizing Problems, while RD and PS were negatively correlated with the YSR subscales. As for the character dimension, SD and CO showed significant negative correlations with Total Problems, Internalizing Problems and Externalizing Problems while ST did not. This trend appeared in both males and females (Tables 3 & 4), respectively, except that HA did not show any significant association with Externalizing Problems in males (Table 3).

**Table 2:** Correlation coefficients between subscales of the Youth Self-Report and Junior Temperament and Character Inventory.

	Junior Temperament and Character Inventory						
	NS	HA	RD	PS	SD	CO	ST
Youth Self Report							
Total Problems	.353^**^	.411^**^	−.242^**^	−.366^**^	−.465^**^	−.336^**^	.008
Internalizing Problems	.127^**^	.525^**^	−.268^**^	−.271^**^	−.469^**^	−.232^**^	.024
Externalizing Problems	.497^**^	.132^**^	−.166^**^	−.323^**^	−.266^**^	−.396^**^	−.074
Anxious/Depressed	.134^**^	.546^**^	−.118^**^	−.221^**^	−.482^**^	−.173^**^	.076^*^
Withdrawn/Depressed	−.009	.510^**^	-.473^**^	−.291^**^	−.440^**^	−.252^**^	−.058
Somatic Complaints	.189^**^	.227^**^	−.129^**^	−.178^**^	−.228^**^	−.171^**^	.019
Rule-Breaking Behavior	.359^**^	.011	−.193^**^	−.285^**^	−.136^**^	−.315^**^	−.134^**^
Aggressive Behavior	.513^**^	.192^**^	−.123^**^	−.300^**^	−.313^**^	−.391^**^	−.023
Social Problems	.232^**^	.370^**^	−.229^**^	−.306^**^	−.443^**^	−.305^**^	−.022
Thought Problems	.232^**^	.314^**^	−.222^**^	−.232^**^	−.312^**^	−.239^**^	.060
Attention Problems	.332^**^	.416^**^	−.145^**^	−.455^**^	−.531^**^	−.224^**^	.062
Other Problems	.386^**^	.148^**^	−.066	−.218^**^	−.221^**^	−.192^**^	.056

**Notes.**

* *p* < 0.05.

** *p* < 0.01.

*** *p* < 0.001.

Bold represents correlation coefficient greater than 0.4.

NS Novelty-Seeking HA Harm-Avoidance RD Reward-Dependence PS Persistence SD Self-Directedness CO Cooperativeness ST Self-Transcendence

**Table 3:** Correlation coefficients between subscales of the Youth Self-Report and Junior Temperament and Character Inventory in male students (*n* = 365).

	Junior Temperament and Character Inventory						
	NS	HA	RD	PS	SD	CO	ST
Youth Self Report							
Total Problems	.339**	.388**	−.271**	−.336**	−.429**	−.355**	.012
Internalizing Problems	.075	.524**	−.348**	−.248**	−.449**	−.268**	.025
Externalizing Problems	.510**	.091	−.126*	−.297**	−.220**	−.378**	−.036
Anxious/Depressed	.106*	.535**	−.202**	−.187**	−.473**	−.235**	.069
Withdrawn/Depressed	−.063	.520**	−.498**	−.264**	−.416**	−.243**	−.022
Somatic Complaints	.154**	.214**	−.206**	−.190**	−.197**	−.203**	−.005
Rule-Breaking Behavior	.226**	.350**	−.265**	−.279**	−.429**	−.338**	−.032
Aggressive Behavior	.216**	.306**	−.222**	−.207**	−.301**	−.222**	.074
Social Problems	.338**	.410**	−.178**	−.424**	−.493**	−.250**	.043
Thought Problems	.383**	.009	−.141**	−.309**	−.131*	−.318**	−.116*
Attention Problems	.533**	.136**	−.099	−.251**	−.253**	−.371**	.023
Other Problems	.388**	.186**	−.118*	−.222**	−.229**	−.238**	.028

**Notes.**

NS Novelty-Seeking HA Harm-Avoidance RD Reward-Dependence PS Persistence SD Self-Directedness CO Cooperativeness ST Self-Transcendence

Bold represents correlation coefficient greater than 0.4.

**Table 4:** Correlation coefficients between subscales of the Youth Self-Report and Junior Temperament and Character Inventory in female students (*n* = 305).

	Junior Temperament and Character Inventory						
	NS	HA	RD	PS	SD	CO	ST
Youth Self Report							
Total Problems	.376**	.447**	−.222**	−.408**	−.521**	−.310**	.007
Internalizing Problems	.184**	.522**	−.270**	−.311**	−.487**	−.216**	−.014
Externalizing Problems	.514**	.222**	−.146*	−.368**	−.374**	−.408**	−.077
Anxious/Depressed	.160**	.557**	−.114*	−.278**	−.489**	−.129*	.042
Withdrawn/Depressed	.076	.515**	−.445**	−.329**	−.493**	−.251**	−.081
Somatic Complaints	.219**	.233**	−.191**	−.188**	−.246**	−.194**	−.022
Rule-Breaking Behavior	.247**	.407**	−.188**	−.348**	−.473**	−.254**	.004
Aggressive Behavior	.266**	.339**	−.203**	−.268**	−.343**	−.252**	.069
Social Problems	.324**	.425**	−.132*	−.499**	−.582**	−.196**	.083
Thought Problems	.407**	.056	−.097	−.257**	−.212**	−.269**	−.054
Attention Problems	.492**	.275**	−.150**	−.371**	−.402**	−.419**	−.078
Other Problems	.386**	.101	−.009	−.212**	−.212**	−.134*	.094

**Notes.**

NS Novelty-Seeking HA Harm-Avoidance RD Reward-Dependence PS Persistence SD Self-Directedness CO Cooperativeness ST Self-Transcendence

Bold represents correlation coefficient greater than 0.4.

Stepwise regression analyses were performed to further analyze the effects of personality traits on Total, Internalizing and Externalizing Problems. Table 5 represents the results of the full model after gender, school year and all seven personality traits of the JTCI were included in the analysis.

**Table 5:** Stepwise regression with seven Junior Temperament and Character Inventory subscales and demographic variables.

	Unstandardized Coefficients	Standardized Coefficients		*t*	*p*-value
	*B*	*SE*	*β*		
Total Problems					
SD^***^	−0.754	0.159	−0.192	−4.730	<0.001
NS^***^	1.435	0.128	0.355	11.173	<0.001
HA^***^	1.008	0.135	0.295	7.450	<0.001
RD^***^	−0.997	0.171	−0.182	−5.829	<0.001
*R*^2^(*adj*.*R*^2^) = 0.38(0.377), *F* = 102.02, *p* < 0					
Internalizing Problems					
Gender^***^	2.191	0.572	0.124	3.833	<0.001
HA^***^	0.519	0.052	0.401	9.987	<0.001
RD^***^	−0.515	0.071	−0.248	−7.306	<0.001
NS^***^	0.213	0.049	0.139	4.347	<0.001
SD^***^	−0.225	0.061	−0.151	−3.673	<0.001
ST^***^	0.128	0.059	0.070	2.154	0.032
*R*^2^(*adj*.*R*^2^) = 0.379(0.374), *F* = 67.499, *p* < 0					
Externalizing Problems					
Gender^***^	−1.927	0.441	−0.139	−4.371	<0.001
NS^***^	0.501	0.040	0.416	12.512	<0.001
CO^***^	−0.271	0.047	−0.203	−5.820	<0.001
SD^***^	−0.170	0.039	−0.145	−4.340	<0.001
*R*^2^(*adj*.*R*^2^) = 0.345(0.341), *F* = 87.425, *p* < 0					

**Notes.**

* *p* < 0.05.

** *p* < 0.01.

*** *p* < 0.001.

NS Novelty-Seeking HA Harm-Avoidance RD Reward-Dependence PS Persistence SD Self-Directedness CO Cooperativeness ST Self-Transcendence

As for the Total Problem, 38% of the variance was explained by the regression model. High NS and high HA were risk factors for Total Problems and high SD and high RD were protective factors for overall behavior problems. Gender showed a contrasting effect on Internalizing and Externalizing Problems, and Internalizing Problems score was higher in female students while Externalizing Problems score was higher in male high school students.

In regard to the Internalizing Problems, the regression model accounted for 37.9% of the variance. High NS, high HA, high ST and the female gender were risk factors for Internalizing Problems, while high SD and high RD were protective factors. The regression model explained 34.5% of the variance in predicting Externalizing Problems. High NS and the male gender were risk factors for Externalizing Problems, while high SD and high CO were protective factors.

### Latent profiles analysis with nine YSR subscale scores

**Figure 1:**
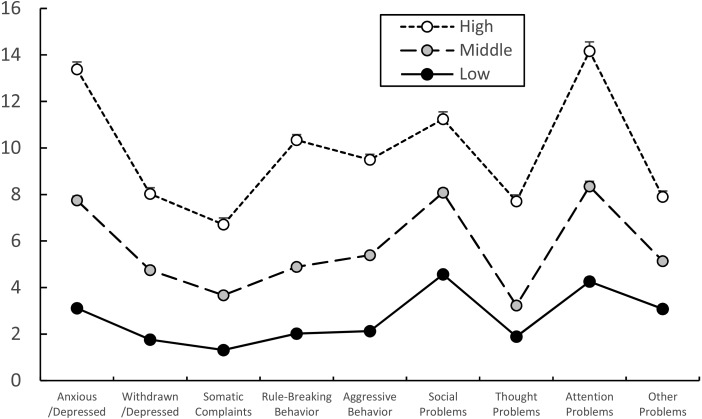
Estimated nine YSR subscale scores of high, middle, and low behavior problem groups. The Youth Self-Report (YSR) subscale profiles of the high, middle and low behavior problem groups were significantly different (flatness with Greenhouse-Geisser correction, *df* = 6.078, *F* = 45.931, *p* < 0.001; parallelism with Greenhouse-Geisser correction, *df* = 12.156, *F* = 30.149, *p* < 0.001). Nine YSR subscales of the three behavior problem groups were significantly different from each other using ANCOVA with gender as the covariate. The estimated value of nine YSR raw scores was calculated with gender as the covariate and used as Y-axis. Data are shown as estimated means and standard errors.

Latent profile analysis identified three latent behavior problem groups as the best solution (Table 6) after controlling the effect of gender, and YSR subscale scores of three (high, middle, and low) latent groups were presented in Fig. 1. The three-latent group model (3-class solution) fit the current data significantly better than a 2-class solution, and was not worse than a 4-class solution considering the smaller BIC and adjusted BIC, the smaller *p* value of VLMR and LMR and the greater entropy. All nine YSR subscale scores of the high, middle, and low behavior problem groups in current study exhibited a decreasing order as shown in Fig. 1. The number of participants of the high, middle, and low groups in regard to behavior problems were 70, 235, and 365, respectively.

**Table 6:** Results of Latent Profile Analysis with gender as covariate.

	BIC	adj.BIC	VLMR*p*	LMR*p*	BLRT*p*	Entropy
2 class	29616.64	29524.563	<0.0001	<0.0001	<0.0001	0.9300
3 class	28979.392	28855.564	0.0446	0.0460	<0.0001	0.8860
4 class	28741.791	28586.212	0.3298	0.3336	<0.0001	0.8920
5 class	28636.385	28449.056	0.3245	0.3273	<0.0001	0.8880

**Notes.**

BIC Bayesian Information Criteria adj.BIC Sample-Size Adjusted BIC VLMR*p* Vuong-Lo-Mendell-Rubin Likelihood Ratio test *p*-value LMR*p* Lo-Mendell-Rubin Adjusted LRT *p*-value BLRT*p* Parametric Bootstrapped Likelihood Ratio *p*-value

### Differences of YSR and JTCI subscales among three latent behavior problem groups using profile analysis and ANCOVA

The significant differences in the YSR subscale profile among the three latent behavior problem groups were analyzed with profile analysis (Fig. 1), and there were significant differences between the three behavior problem groups. The YSR subscales of the three behavior problem groups were not flat (Greenhouse-Geisser correction, *df* = 6.078, *F* = 45.931, *p* < 0.001), and the interaction of three groups was significantly different in regard to parallelism (Greenhouse-Geisser correction, *df* = 12.156, *F* = 30.149, *p* < 0.001). There were significant differences in nine YSR subscales of Anxious/Depressed (*F* = 533.559, *p* < 0.001), Withdrawn/Depressed (*F* = 283.200, *p* < 0.001), Somatic Complaints (*F* = 182.751, *p* < 0.001), Social Problems (*F* = 577.616, *p* < 0.001), Thought Problems (*F* = 523.450, *p* < 0.001), Attention Problems (*F* = 259.036, *p* < 0.001), Rule-Breaking Behavior (*F* = 184.601, *p* < 0.001), Aggressive Behavior (*F* = 307.468, *p* < 0.001) and Other Problems (*F* = 186.481, *p* < 0.001) based on ANCOVA results. Nine YSR subscale scores of the three behavior problem groups were significantly different from each other as shown in Fig. 1.

**Figure 2:**
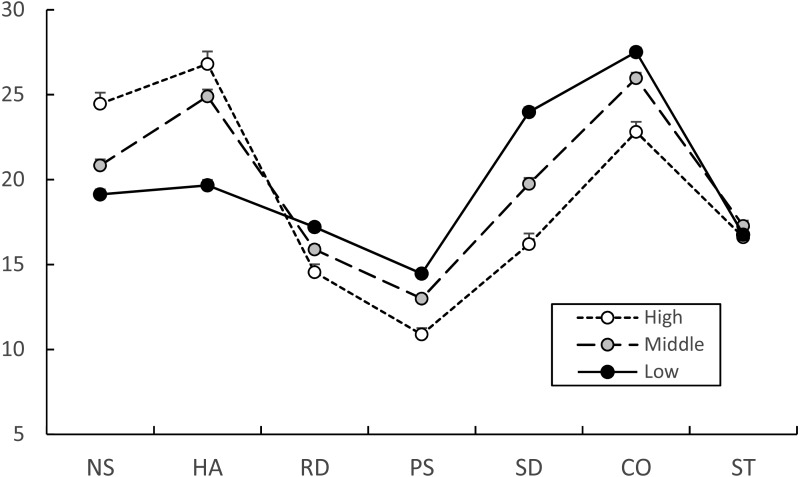
Estimated Junior Temperament and Character Inventory subscale scores of high, middle, and low behavior problem groups. The JTCI subscale profiles of the high, middle and low behavior problem groups were significantly different (flatness with Greenhouse-Geisser correction, *df* = 3.949, *F* = 71.200, *p* < 0.001; parallelism with Greenhouse-Geisser correction, *df* = 7.897, *F* = 52.974, *p* = 0.001). The JTCI subscales, excluding ST, of the three behavior problem groups were significantly different from each other using ANCOVA with gender as the covariate. The estimated value of six JTCI raw scores was calculated with gender as the covariate and used as Y-axis. Data are shown as estimated means and standard errors. NS, Novelty-Seeking; HA, Harm-Avoidance; RD, Reward-Dependence; PS, Persistence; SD, Self-Directedness; CO, Cooperativeness; ST, Self-Transcendence.

The significant differences on the JTCI subscale profile among the three behavior problem groups were analyzed with profile analysis (Fig. 2), and there were significant differences between three latent groups. The JTCI personality profile of the three behavior problem groups was not flat (Greenhouse-Geisser correction, *df* = 3.949, *F* = 71.200, *p* < 0.001), and the interaction of three groups was significantly different in regard to parallelism (Greenhouse-Geisser correction, *df* = 7.897, *F* = 52.974, *p* = 0.001). There were significant differences in the six JTCI subscales of NS (*F* = 29.334, *p* < 0.001), HA (*F* = 73.850, *p* < 0.001), RD (*F* = 17.135, *p* < 0.001), PS (*F* = 43.357, *p* < 0.001), SD (*F* = 89.575, *p* < 0.001) and CO (*F* = 28.356, *p* < 0.001), but not ST (*F* = 1.048, *p* = 0.351). High, middle, and low latent groups of NS, HA were in decreasing order, while those of RD, PS, SD and CO were in increasing order with significant differences as shown in Fig. 2.

## Discussion

This study recruited 670 Korean high school students, and measured behavior problems and personality characteristics with the YSR and JTCI to investigate which personality traits and profiles explain behavior problems in adolescents.

Demographic characteristics showed significant differences in the YSR and JTCI subscale scores between male and female high school students (Table 1). As for behavioral problems, female students showed higher scores on Internalizing Problems including Anxious/Depressed and Withdrawn/Depressed, and male students evidenced higher scores in Externalizing Problems such as Rule-Breaking Behavior (Table 1) which is consistent with results of previous study (Oh & Lee, 1990). Gender was found to account for the type of behavior problem (Table 5) such that the male gender explained Externalizing Problems and the female gender delineated Internalizing Problems. As for personality traits, females exhibited higher RD, CO and ST scores compared to male students, which are consistent with previous studies (Lee, Choi & Chae, 2017a; Lee et al., 2014; Zohar et al., 2018).

The effects of temperament and character traits on YSR Total, Internalizing and Externalizing Problems were analyzed with stepwise regression analysis (Table 5). As for the JTCI temperament domain with a biological basis, NS and HA were risk factors and RD was a protective factor for YSR Total and Internalizing Problems, and NS was a risk factor for YSR Externalizing Problems. These results are in accordance with previous studies which showed that high HA and low RD is associated with Total and Internalizing Problems (Copeland et al., 2004; Hemphälä, Gustavsson & Tengström, 2013; Kim, 2013; Kim et al., 2006; Lim, Choi & Choi, 2013; Rettew et al., 2006) and high NS is related to Externalizing Problems (Copeland et al., 2004; Ha & Kwon, 2016; Hemphälä, Gustavsson & Tengström, 2013; Kim & Lee, 2016; Kim et al., 2006; Rettew et al., 2006).

Although NS significantly explained Internalizing Problems unlike previous studies, considering that NS and Internalizing Problems showed a negative correlation after controlling for Externalizing Problems using partial correlation analysis, this result may have stemmed from comorbid Internalizing and Externalizing Problems. It was reported that high NS shows a relation with development of delinquent behavior and antisocial personality (Schmeck & Poustka, 2001), and NS was the best predictor of highly delinquent behavior (Tremblay et al., 1994). In other words, such results are similar to a study examining social anxiety disorder in an outpatient sample (Kashdan & Hofmann, 2008) indicating the need for a more careful interpretation in regard to comorbidity of adolescent behavior problems.

As for the JTCI character domain, high SD may be a protective factor for YSR Total, Internalizing and Externalizing Problems along and high CO for YSR Externalizing Problems in adolescents. This result is consistent with previous studies (Cloninger, Svrakic & Przybeck, 1993; Copeland et al., 2004; Hemphälä, Gustavsson & Tengström, 2013; Kim, 2013; Kim et al., 2006; Lim, Choi & Choi, 2013; Rettew et al., 2006) which reported that high SD and high CO explains resilience to behavior problems. The JTCI RD and CO might be crucial factors in determining the expression and progression of behavior problems considering its theoretical correlation with agreeableness and protective effects of agreeableness in Externalizing Problems (Laursen & Richmond, 2013; Slobodskaya & Akhmetova, 2010). In summarizing the relationship between behavior problems and personality traits, we can conclude that high HA, high NS and low RD of temperament domain and low SD and low CO of character domain may be a particular vulnerability factor for adolescent behavior problems.

Behavior problems in real life evolve through complex interactions between diverse factors, rather than through several problematic symptoms individually influenced by one or two personality traits. Therefore, the personality characteristics of an individual must be analyzed as a multi-dimensional and complex system using person-centered Latent Profile Analysis (LPA). The diagnosis, treatment, and prevention of behavior problems should be practiced with this whole-person perspective in mind as suggested previously (Eley et al., 2016; Lee, Choi & Chae, 2017a).

We performed LPA analysis on nine subscales of the YSR to extract distinctive latent profile groups (Table 6), and found three latent groups consisting of high, middle, and low behavior problem groups. These three behavior problem groups were distinctively and significantly different from each other in regard to YSR behavior problem profiles and also the nine YSR subscale scores (Fig. 1). There also were significant differences among the three behavior problem groups in regard to JTCI personality profiles and also the JTCI subscales scores with the exception of ST (Fig. 2).

These results provided information regarding the temperament and character profiles of community adolescents which may predict matured personality, positive affect, psychological well-being, and life satisfaction in adulthood (Garcia, 2011; Garcia et al., 2013b; Garcia & Moradi, 2012; Moreira et al., 2014). In current study, the high behavior problem group showing a vulnerable personality profile was characterized by high HA and NA and low RD, PS, SD and CO. However, the low behavior problem group showing a resilient personality profile was characterized by low HA and NA and high RD, PS, SD and CO which is in line with the healthy and resilient personality profile reported in previous studies (Eley et al., 2016; Lee, Choi & Chae, 2017a; Moreira et al., 2014) as Dependable temperament and Matured character profile (Zohar et al., 2018). The temperament traits of high NS and/or high HA in combination with character traits of low SD were repeatedly demonstrated as a major risk factor for many psychopathological conditions (Fassino et al., 2004; Lee, Choi & Chae, 2017a; Lilenfeld, 2010).

The temperament complex of high NS, high HA and low RD (NHr) represented the high behavior problem group in the current study, and the most vulnerable personality profile related to psychological immaturity (Basoglu et al., 2011; Cloninger, 1987; Lee et al., 2014) was characterized by an Explosive temperament profile which possesses dynamic psychological energy secondary to a simultaneous co-existing mixture of approach (high NS) and avoidance (high HA) behaviors (Cloninger, 1987; Lee et al., 2014). Individuals with an Explosive temperament profile tend to be bold and daring when others hesitate and/or behave according to the particular situation. There is also a high probability of conflicts in interpersonal relationships since they may express their impulsivity and emotions without filtering, and may lack empathic capability secondary to their temperament trait of low RD.

On the contrary, an individual with a low NS, low HA and high RD (nhR) representing the low behavior problem group in this study manifests a Reliable temperament profile which is potentially the least vulnerable to psychological immaturity (Cloninger, 1987; Lee et al., 2014). Individuals with Reliable and Dependable (low NS, low HA, high RD and high PS) temperament profile are calm and careful, and are generally optimistic, pleasant, and conscientious. They are compassionate and express soft and warm emotions, while not being easily angered, annoyed nor excited.

In these ways, when analyzing psychological characteristics from the perspective of interacting complexes, rather than as a collection of individual factors, we may find that the immature coping style of the Explosive temperament profile (Cloninger, 1987; Lee et al., 2014) could trigger maladaptive response styles to daily life stress, and potentially exacerbate behavior problems, and the Matured character profile of high SD and CO provides cognitive self-regulation of their behavior and adaptive responses to stress (Moreira et al., 2014; Zohar et al., 2018).

Adolescence is an important period for the development of autonomous identity, social relationships, and values and goals shaping their lifestyle (Erikson, 1968; Garcia et al., 2013b), and psychological maturity measured as high SD and CO is a predictor of appropriate adaptation to society and/or ability to bounce back from lifespan challenges (Cloninger, Salloum & Mezzich, 2012a; Eley et al., 2013; Moreira et al., 2014). It is a period of identity formation showing dramatic transition from external inhibition on emotional drive by parents, teachers and guardians to cognitive and intentional self-regulation based on their own goals and values (Erikson, 1968; Moreira et al., 2014; Zohar et al., 2018).

Adolescents and adults with high SD and CO character experience more frequent positive emotions and life satisfaction throughout their lifetime, and less psychosocial dysfunction and behavioral disorders which reportedly increase dramatically during adolescence (Garcia, Anckarsäter & Lundström, 2013a; Garcia et al., 2013b). The matured adults demonstrate higher executive functioning such as response inhibition, attention, long-range planning and organization, and flexible regulation of impulses and employment of cognitive emotional strategies emerge and grow by adolescence (Garcia et al., 2013b).

Since the most effective preventive intervention for behavior problems may be accomplished by enabling matured coping to stress through promotion of character development both at home and school (Eley et al., 2013; Eley et al., 2016; Moreira et al., 2014; Zohar et al., 2018), emphasis should be placed on fostering personal integrity, self-esteem, self-acceptance, responsibility, relatedness and effectiveness in society (Cloninger, 2006; Magen, 1998) which the JTCI SD and CO character traits accentuate.

Children with high RD, PS, SD and CO were shown to exhibit high intelligence and academic achievement (Copeland et al., 2004), self-discipline in adolescents was reported to be a better predictor of academic performance than intellectual functioning in school (Duckworth & Seligman, 2005; Moreira et al., 2012), and matured adolescents exhibited higher level of positive affect, life satisfaction, social support and subjective health (Moreira et al., 2014). Educating adolescents receiving and providing help as peer counselors may be an exemplary intervention which is useful for strengthening a sense of purpose and worth, feelings of usefulness, and satisfying interaction with others (Garcia et al., 2013b; Magen, 1998).

This study may hold a number of limitations in regard to generalization. First, participants in the current study were high school students from a metropolitan area, and the ratio of well-adjusted to problematically adjusted students in addition to rural or urban characteristics (Eley et al., 2016) may have affected the outcome. Although the sample size was sufficiently large enough to dampen potential biases, it will be needed to be validated in diverse aspects to generalize the findings.

Second, the current study examined the personality characteristics of high school students who exhibits relatively stable temperament and character and can complete reliable self-report measures. Considering that behavior problems and coping mechanism in response to stress situations develop in complex interaction with temperament, character, environment, and learning (Cloninger, Salloum & Mezzich, 2012a; Eley et al., 2013), the findings of the current study with high school students should be also be replicated in middle school and elementary school students as well. This would allow for early and proactive preventive interventions through reviewing lifelong developmental perspectives on personality and psychopathology.

## Conclusion

The current study confirmed previous reports (Eley et al., 2013; Eley et al., 2016; Lee, Choi & Chae, 2017a) that high NS and HA and low RD temperaments and low SD and CO characters are risk factors for behavior problems in adolescents both as individual traits and as a complex profile (Moreira et al., 2014; Zohar et al., 2018). The results would contribute to studies examining effects of personality on behavior problems and benefits of improving resilience to stress and nurturing lifespan psychological well-being by developing matured personality (Eley et al., 2016).

##  Supplemental Information

10.7717/peerj.6106/supp-1 Supplemental Information 1 Dataset Data for analyzing relation between TCI and YSR subscales are provided.

## Additional Information and Declarations

### Data Availability

The following information was supplied regarding data availability: The raw data are provided in the Supplemental Information 1.
